# Enhanced Reliability of the Evaluation of Fertility Traits in Pura Raza Española Horses Using Single-Step Genomic Best Linear Unbiased Prediction

**DOI:** 10.3390/genes16050562

**Published:** 2025-05-09

**Authors:** Chiraz Ziadi, Mercedes Valera, Nora Laseca, Davinia Perdomo-González, Sebastián Demyda-Peyrás, Arancha Rodríguez-Sainz de los Terreros, Antonio Molina

**Affiliations:** 1 Departamento de Genética, Universidad de Córdoba, 14071 Córdoba, Spain; z72zizic@uco.es (C.Z.); ge2depes@uco.es (S.D.-P.); 2 Departamento de Agronomía, Universidad de Sevilla, 41013 Sevilla, Spain; mvalera@us.es (M.V.); nlaseca@us.es (N.L.); 3 Real Asociación Nacional de Criadores de Caballos de Pura Raza Española (ANCCE), 41014 Sevilla, Spain; arancha@lgancce.com; 4 Departamento de Producción Animal, Universidad Complutense de Madrid, 28040 Madrid, Spain; daperdom@ucm.es

**Keywords:** equine, fertility, reliability, single-step GREML, SNP genotyping

## Abstract

**Background/Objectives:** By simultaneously integrating both genotyped and non-genotyped animals into genetic evaluation, the single-step genomic BLUP method enhanced the accuracy of genetic assessments. This study aimed to compare the increase in prediction reliability (R^2^) between restricted maximum likelihood (REML) and single-step genomic REML (ssGREML) in the Pura Raza Española (PRE) horse breed. **Methods:** The dataset comprised reproductive records for seven fertility traits from 47,502 females, with a total of 57,316 animals represented in the pedigree. A total of 4009 animals were genotyped using the EQUIGENE 90K SNP array, and 71,322 SNPs were retained for analysis after quality control. Genetic parameters were estimated using a multivariate model with the BLUPF90+ v2.60 software. **Results:** Heritability estimates were similar between REML and ssGREML, ranging from 0.07 for IF12 to 0.349 for ALF. An increase in R^2^ was observed with ssGREML compared to REML across all traits, with overall gains ranging from 2.20% to 3.71%. Among genotyped animals, R^2^ values ranged from 17.81% to 24.04%, while significantly lower values (0.80% to 2.34%) were observed in non-genotyped animals. Notably, individuals with low initial R^2^ values under the REML approach exhibited the most significant gains using ssGREML. This improvement was particularly pronounced among stallions with fewer than 40 controlled foals. **Conclusions:** Our results demonstrated that incorporating genomic data improves the reliability of genetic evaluations for mare fertility in PRE horses.

## 1. Introduction

Maintaining high fertility is essential to a mare’s economic value, as successful breeding and reproduction are crucial for sustaining a healthy and profitable equine operation. Furthermore, the birth of a foal represents not only the continuation of a valuable bloodline but also a significant investment of time and resources [1]. Therefore, obtaining reliable genetic parameters and achieving high accuracy in estimated breeding values for economically important traits, particularly those with low heritability, such as fertility, is crucial for successful genetic improvement programs.

The Pura Raza Española (PRE) is a native Spanish equine breed, officially recognized since the establishment of its studbook in 1912. It has an active population of 282,066 horses, primarily located in Spain but also distributed across 67 other countries [2]. The breeding program is managed by the Royal National Association of Spanish Horse Breeders (ANCCE), with primary objectives focused on improving morphology, conformation, and functionality, while maintaining genetic diversity, reducing inbreeding levels, and preserving the breed’s genetic heritage [3]. Nevertheless, many PRE breeders continue to follow an in-line breeding system, which has been shown to increase inbreeding and contribute to inbreeding depression, ultimately leading to a decline in reproductive efficiency [4]. Moreover, fertility is a complex polygenic trait with low heritability [5], influenced by various environmental and management factors [6].

For these reasons, there has been growing interest in recent years in analyzing various reproductive traits and understanding the genomic mechanisms underlying fertility in PRE mares [7,8,9].

To date, PRE breeding improvement programs have relied on mixed models based on the best linear unbiased prediction (BLUP) methodology. However, advancements in SNP genotyping technologies, along with the recent development of a high-density chip specific to this breed, have provided sufficient genomic information to enhance our understanding of complex traits such as fertility.

Genomic selection has emerged as a powerful tool for accelerating genetic improvement in breeding programs worldwide [10]. Several approaches have been developed to incorporate genomic information into genetic evaluation models. The genomic best linear unbiased prediction (GBLUP) approach [11] uses the realized relationships between animals through the genomic relationship matrix, with the relatedness among animals estimated using genomic markers. However, the practical implementation of genomic data still faces challenges, including genotyping costs and the lack of phenotypic or genotypic information for a large number of animals. In this context, the single-step genomic best linear unbiased prediction (ssGBLUP) approach has been proposed for estimating genomic breeding values [12,13]. This methodological approach allows for the simultaneous use of all available information from both genotyped and non-genotyped relatives, along with phenotypic data [14].

ssGBLUP has been shown to provide greater reliability than other methods of genetic merit evaluation across various species [15,16,17]. However, there is limited evidence and documentation regarding improvements and changes in fertility traits, particularly in the equine species, since the introduction of genomic evaluations. In our previous study [18], the ssGBLUP approach resulted in a substantial improvement in the reliability of genomic prediction for morphological traits in PRE horses, making its extension to other traits within this breed promising.

Therefore, the aim of this study was to compare the reliability of genomic breeding values for fertility traits between the traditional REML and the single-step genomic REML approaches in the Pura Raza Española, the most important horse breed in Spain.

## 2. Materials and Methods

### 2.1. Records and Pedigree

Data on Pura Raza Española (PRE) fertility traits were provided by the Royal National Association of Spanish Horse Breeders (ANCCE). The reproductive data included records of 47,502 females across seven fertility traits, calculated as described by Perdomo-González et al. [4]. The traits under consideration included age at first foaling (AFF) in years, age at last foaling (ALF) in years, average interval between foalings (AIF) in months, total number of foalings (FN), average interval between first and second foalings (IF12) in months, productive life (PL) in years, and reproductive efficiency (RE). PL was calculated individually for each mare from the age at first foaling and the age at which the mare was culled from the breeding herd, or the age at her last foaling if she had not completed her productive life. RE represents the relationship between the total number of foalings of a mare and the optimal number of foalings she could achieve over her lifetime. For each mare, the optimal number of foalings was determined based on the age at first foaling and the age of reproductive culling or last recorded foaling, in the case she is still reproductively active. This calculation assumed an optimal foaling frequency of one foaling per year, starting from her initial foaling [7].

To obtain the genealogical kinship matrix, the ancestors of the controlled mares were traced back through all generations, resulting in a total of 57,316 animals, spanning eight complete generations and eleven equivalent generations. 

### 2.2. Genomic Information

Genotypic data comprised 4009 individuals genotyped with the EQUIGENE 90K SNP array, including over 90,000 SNPs per individual. Sample selection of the genotyped horses was performed, aiming to capture the maximum variability of the population and to include the most representative horses of the population, and was based on the low average relatedness among individuals. These horses came from more than 600 different studs and were chosen to reflect the variability of the PRE breed. The genomic data were filtered for monomorphic SNPs, SNPs and individuals with a call rate lower than 95%, and SNPs with a minor allele frequency less than 0.05. After quality control, 71,322 informative SNPs were used in the analysis. SNP and sample quality control were performed using PLINK v1.9 [19].

### 2.3. Statistical Analysis

The significance of the fixed effects for fertility traits was determined using the ‘GLM2’ package [20] in the R statistical environment V4.4.0 [21]. All the fixed effects had a significant effect at the 0.05 significance level. The descriptive statistical parameters (mean, standard deviation, minimum, maximum, and coefficient of variation) of the studied traits were calculated using the R statistical environment V4.4.0 [21].

The genetic evaluation was performed using a multivariate model as follows:

y=Xb+Za+e

where y is the vector of phenotypes; b is the vector of fixed effects, including inbreeding as a linear covariate, age at the mare’s last foaling as a linear covariate (except for AFF, ALF, and IF12), ancestral origin (two classes: exclusively Hispanic origin—phenotypically with gray coat color—and with influence of Central European and Arabian breeds—phenotypically with non-gray coat color), geographic stud zone (three classes: Spain, the rest of Europe, and the rest of the world), and average stud size in the decade of the mare’s first foaling (three classes: less than 3 foals born per year, between 3 and 9 foals born per year, and more than 9 foals born per year). a is the random additive genetic effect, and e is the random residual effect. X and Z are incidence matrices relating observations to fixed and random additive genetic effects, respectively. It was assumed that

$$E\left[y\right]=Xb,$$

where $E\left[y\right]$ is the expected value of the vector of observed phenotypic records y, and b is the vector of fixed effects as described above.

The variance structures of a and e were assumed to be as follows:

$$var\left[\begin{array}{c} a\\ e \end{array}\right]=\left[\begin{array}{cc} A⊗G_{0} & 0\\ 0 & I⊗R_{0} \end{array}\right]$$

where a is the vector of the additive genetic effect, e is the vector of the residual effect, A is the numerator relationship matrix, $G_{0}$ is the covariance matrix of the additive genetic effects, and $R_{0}$ is the covariance matrix of error among traits.

For ssGREML, the numerator relationship matrix A in the equation above was replaced by H, as defined by Legarra et al. [22]. The H matrix was derived by integrating A with the genomic relationship matrix G calculated using VanRaden [11] as follows:

$$G=0.95\frac{ZZ\prime }{2\sum_{i=1}^{n}p_{i}(1-p_{i})}+0.05A$$

where n is the number of SNP markers, $p_{i}$ is the allele frequency of marker i, A is the pedigree relationship matrix, and Z is a centered incidence matrix of SNP markers.

Variance components and breeding values used in the REML and ssGREML models were estimated using the restricted maximum likelihood approach with BLUPF90+ v2.60 software [23].

## 3. Results and Discussion

### 3.1. Phenotypic Values

Descriptive statistics for each fertility trait in the PRE breed are shown in Table 1. The average phenotypic values were 63.83 months ± 32.99 for AFF, 172.74 months ± 66.85 for ALF, 19.89 months ± 10.13 for AIF, 6.36 ± 4.33 for FN, 18.97 months ± 13.44 for IF12, 136.27 months ± 63.49 for PL, and 47.83% ± 19.41 for RE. The coefficients of variation ranged from 34.15% (AFF) to 71.32% (FN). Our mean values were similar to those observed in previous studies of the same breed [7,9].

In the study by Gómez et al. [7] on other Spanish horse breeds, including Arab horses, Spanish Sport Horses, Anglo-Arabs, and Spanish Trotters, the average values of AFF and AIF were significantly higher than those of PRE mares. These findings suggest the precocity (earlier sexual maturity) and reproductive superiority of PRE mares compared to these breeds, allowing PRE females to conceive at a younger age. In addition to genetic factors, it is important to emphasize that the age at first mating is also determined by management practices and decisions—such as excluding the animal from reproduction while it is competing, feeding, healthcare, and reproductive strategies—which can differ significantly between the PRE breed and other breeds.

### 3.2. Estimation of Variance Components and Heritability

Historically, routine genetic evaluations in the PRE breed have been based on the BLUP methodology, utilizing only phenotypic and pedigree information. However, in recent years, genotyping has been introduced in the PRE breed, alongside the development of a breed-specific chip designed to maximize the number of highly informative markers (with a minor allele frequency close to 0.5). This advancement enabled the application of genomic evaluation for economically important traits in the breed.

The variance components and heritability (h^2^) estimates were almost similar in the REML and ssGREML analyses, as shown in Table 2. The estimates of h^2^ ranged from low to moderate, varying from 0.070 ± 0.007 (IF12) to 0.355 ± 0.010 (ALF).

Our estimates closely align with the h^2^ observed by Laseca et al. [24], who utilized A and H matrix relationships to estimate inbreeding depression in the same breed. Significant differences were observed when comparing our values to those reported by Gómez et al. [7] for other Spanish breeds, particularly the Spanish Sport Horse, which had heritability estimates of 0.32 for AFF, 0.25 for ALF, 0.30 for AIF, 0.30 for IF12, and 0.20 for PL. In the Anglo-Arab Horse, h^2^ values ranged from 0.04 (IF12) to 0.27 (AIF), while in the Spanish Trotter Horse, they varied from 0.09 (AFF and ALF) to 0.42 (AIF). Karlau et al. [25] reported lower values for AFF (0.16 ± 0.014) and RE (0.11 ± 0.013) and higher values for ALF (0.19 ± 0.015) in Criollo Argentino horses compared to our estimates.

The differences in estimates between these breeds may be due to the different models used, the quality of data and pedigree records, the number of available phenotypes, and the level of data connectedness [26]. These factors are all known to impact the estimation of genetic parameters. Additionally, it is important to note that management practices (feeding, health, reproductive management, etc.) differ significantly between the PRE breed and other breeds.

These substantial differences support the hypothesis that a genetic component influences the reproductive phenotypes of mares, contrary to the prevailing notion that fertility traits in mares are predominantly shaped by environmental factors and exhibit low heritability. The latter perspective suggests slower genetic progress compared to other economically important traits, such as morphological and performance traits.

Since fertility traits have low reliability in genetic values (being expressed in only one sex, having generally low heritability, and having often limited performance data, among other factors), there is growing interest in obtaining highly reliable assessments as quickly as possible to achieve significant genetic gain in the breed in the shortest period of time. Additionally, these traits have been shown to be highly sensitive to inbreeding depression, which further complicates genetic progress. Several studies have demonstrated that fertility traits are among the most affected by inbreeding depression in various species [24,27,28]. Furthermore, fertility traits are influenced by interactions between multiple genes and genetic pathways [8], which can make it difficult to identify specific genes or genetic markers associated with the traits analyzed. Therefore, improving female fertility in horses is particularly challenging, as it is influenced by factors that often complicate the direct identification of the underlying causes related to the animals themselves [29]. Despite these limitations, the improvement of fertility traits in PRE mares could be achieved through better recording of reproductive information, selective breeding, and the use of advanced reproductive technologies, such as artificial insemination and embryo transfer [9]. Furthermore, these authors demonstrated that using selection indices theory, a substantial increase in selection response occurs when both morphological and fertility traits are jointly included as selection criteria, with the objective of increasing female reproductive efficiency.

### 3.3. Comparison of Reliability Between REML and ssGREML

The mean prediction reliability for fertility traits under the REML and ssGREML methodologies is presented in Table 3. The results of this study showed that the overall mean reliabilities using REML, calculated for all animals in the pedigree, ranged from 0.244 for IF12 to 0.487 for ALF. Meanwhile, the overall mean reliabilities obtained with ssGREML varied from 0.249 for IF12 to 0.498 for ALF. This indicates that the reliabilities of breeding values obtained from ssGREML were higher than those from the classic REML model for all traits. The increases in reliability from ssGREML compared to REML, expressed as percentage differences, were 3.00%, 2.32%, 2.98%, 3.71%, 2.20%, 3.56%, and 3.13% for AFF, ALF, AIF, FN, IF12, PL, and RE, respectively.

Furthermore, the R^2^ values from the REML and ssGREML approaches were evaluated based on various criteria, including sex, number of foals per sire, genotype status (genotyped vs. non-genotyped), and initial REML reliability. The corresponding results are presented in Table 4. The gain in reliability was distinctly higher in genotyped animals (ranging from 17.81% to 24.04%) compared to non-genotyped animals (0.80% to 2.34%). Additionally, the gain was greater in animals with lower initial REML reliability (2.30% to 3.84%), while those with higher initial REML R^2^ did not experience any improvement. The increase in R^2^ was higher in stallions (ranging from 3.89% to 5.24%) than in mares (1.87% to 3.44%). Among stallions, those with fewer than 40 controlled foals showed greater gains (3.81% to 3.94%) compared to those with more than 40 controlled foals (0.51% to 3.16%) across all traits.

**Figure 1:**
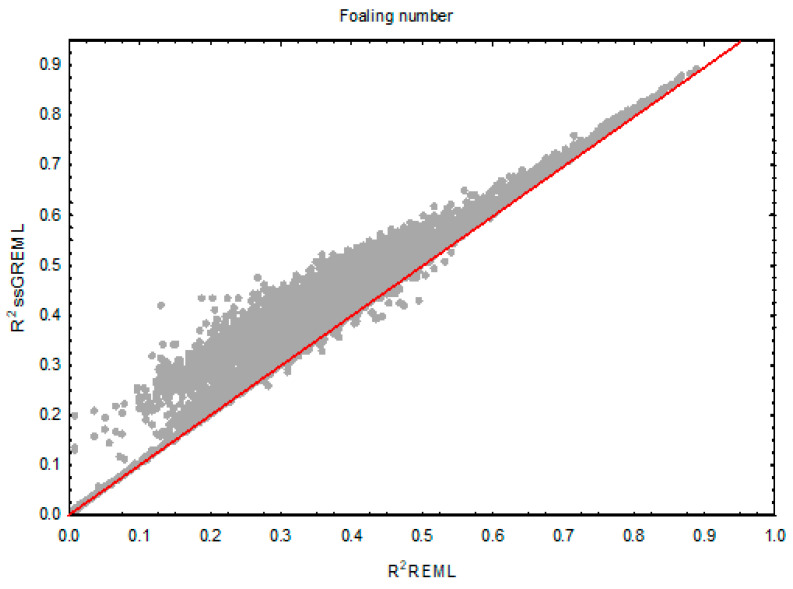
Comparison of reliabilities between REML (R^2^ REML) and ssGREML (R^2^ ssGREML) methods for foaling number in the Pura Raza Española horse breed. The red line corresponds to the linear regression fit between R^2^ values estimated by REML and ssGREML.

As an illustrative example, Figure 1 shows the comparison between the reliabilities obtained from REML and ssGREML for the foaling number (FN) trait, which exhibited the greatest overall increase in R^2^. It can be seen that animals with low REML reliability experienced a larger improvement in reliability.

Regarding horses, Vosgerau et al. [30] reported that incorporating genotype information led to an increase in the R^2^ of genomic predictions for both genotyped and non-genotyped animals, with prediction accuracy improving as the number of phenotyped and/or genotyped descendants increased. In a previous study, Haberland et al. [31] observed that the additional increase in accuracy obtained from GEBVs was small compared to traditional EBVs for animals with a large number of progeny records, which is consistent with our findings.

Genotype information was used in the analysis of fertility traits and inbreeding depression in PRE by Laseca et al. [24], but these authors did not analyze its impact on genetic parameters and reliability. Additionally, the number of genotyped animals was limited to 1018 mares. In a preceding study on the estimation of genetic parameters using both methodological approaches, with the population of genotyped animals extended to include a total of 2916 animals, our research [18] compared the R^2^ of morphological traits. The results showed that the R^2^ estimated with pedigree was lower than that estimated using ssGREML, with the overall increase in R^2^ ranging from 1.56% for the direction of the hock rear view to 13.30% for the angle of the croup.

The application of genomic selection in horse breeding remains scarce and is limited to a few traits and a small number of breeds. Vosgerau et al. [30] reported that the ssGBLUP model performed better than the pedigree-based BLUP model for height at withers in German Warmblood horses, with a gain of 8.57%. Similarly, in the study by Haberland et al. [31], the integration of genomic information significantly increased the reliability of breeding value estimates, particularly for young horses (from 0.27 to 0.54). However, to the best of our knowledge, no studies have been conducted on the estimation of genetic fertility parameters in mares using genomic information, making the comparison of our results with other studies impossible.

In dairy cattle, the first species to include genotype information in genetic evaluations, previous studies have reported that the ssGBLUP method results in higher accuracy compared to the traditional pedigree-based BLUP method [12,32]. Studies in dairy cattle have been expanded to encompass a wide range of traits, including production, fertility, longevity, and health traits. In dairy sheep, the reliability increased to 46.8% [33] and 47.98% [14], while in dairy goats, the increase in accuracy was from 5% to 7% for milk traits [34]. So far, the single-step genomic evaluation method has not been applied to fertility-related traits in small ruminants.

Single-step GBLUP has emerged as the preferred method for the genetic evaluation of both genotyped and ungenotyped livestock. The observed increase in reliability could be attributed to the additional variation in genomic information captured through Mendelian sampling by a realized relationship matrix [15]. Hence, it was noted that the gain in accuracy from pedigree to genomic predictions can be explained by improved relationships [11,35].

Several factors influence the reliability of genomic evaluation, including the extent and distribution of linkage disequilibrium between markers and quantitative trait loci [35,36], the size of the genotyped population [37,38], and the relationship between individuals in the training and validation data [39]. Additionally, the composition of the reference population [37,38,40], heritability [35,41,42], and the statistical method used to include genomic information [43] significantly impact the prediction accuracy of breeding values.

ssGBLUP has been shown to outperform traditional methods in predicting low-heritability traits, particularly those related to reproduction [44]. In the present study, the R^2^ values obtained using ssGREML generally increased as the trait’s heritability decreased. These findings are consistent with the results reported by VanRaden et al. [45] and Misztal et al. [46]. For lowly heritable traits, such as fertility, a very large genotyped population is required to achieve high accuracy of GEBVs [35,44], which could be a challenge for some breeds where the number of genotyped animals remains limited.

Breeding horses are characterized by a higher generation interval compared to other farm animal breeds [47], which results in lower accuracies of breeding values [48]. However, studies have shown that breeding programs with long generation intervals and traits with low heritability can achieve substantial genetic gain when incorporating SNP genotyping in genetic evaluations [29,49]. These insights support the demand to evaluate the potential of ssGBLUP for horse breeding programs.

Fertility traits are heavily influenced by environmental and management factors [50]. Also, phenotypic improvements in female reproductive performance have been achieved through advances in reproductive management practices and the use of reproductive biotechnologies, such as hormonal synchronization of estrus and ovulation, followed by artificial insemination and embryo transfer. However, in equine species, in general, and the PRE breed in particular, the adoption of these techniques remains limited. Thus, ssGBLUP could enable more informed selection decisions at an earlier age, potentially reducing generation intervals and accelerating genetic progress.

Although the incorporation of genomic information into breeding programs is expected to reduce the rate of inbreeding [51], several studies have reported that genomic selection can actually lead to increased levels of inbreeding in the medium term [52,53,54]. This outcome arises from the rapid generational turnover enabled by the shortened generation interval, as young animals are increasingly used as parents [55]. As genetic variability declines, the potential for future genetic improvement diminishes, and populations may become more vulnerable to diseases and environmental stressors. Therefore, while genomic selection offers significant short-term gains in performance, it must be carefully managed to mitigate the risks of inbreeding and ensure long-term sustainability, especially in breeds that already exhibit high levels of inbreeding, such as the Pura Raza Española.

## 4. Conclusions

The results of this study indicate that the breeding values obtained using single-step genomic methods are more accurate than those derived from pedigree-based information for the genetic evaluation of fertility traits in the Pura Raza Española breed. Nevertheless, considerable challenges remain in incorporating genomic information into routine genetic evaluations, implementing genomic selection, and managing inbreeding with genomic selection in the PRE breed. While improving fertility is a desirable goal in the PRE breed, over-selection for this trait may lead to unintended consequences, such as the neglect of other important traits, including performance, conformation, or temperament—particularly those that may be genetically negatively correlated with fertility. To date, no studies in this species have demonstrated that intense selection for fertility negatively impacts other traits, as has been observed in other species such as pigs and poultry.

## Figures and Tables

**Table 1:** Basic statistics of fertility traits in the Pura Raza Española breed.

Trait	No. Records	Mean	SD	Min	Max	CV (%)
AFF, months	47,477	63.83	32.99	23	1287	51.69
ALF, months	39,620	172.74	66.85	45	481	38.70
AIF, months	39,497	19.89	10.13	6	323	50.94
FN	47,502	6.36	4.33	1	24	68.06
IF12, months	39,504	18.97	13.44	9	323	70.85
PL, months	22,646	136.27	63.49	7	433	46.59
RE, (%)	39,620	47.83	19.41	5.26	150	40.57

SD: standard deviation; CV: coefficient of variation; AFF: age at first foaling; ALF: age at last foaling; AIF: average interval between foalings; FN: total number of foalings; IF12: interval between first and second foaling; PL: productive life; RE: reproductive efficiency.

**Table 2:** Estimates of variance components and heritabilities for fertility traits in the Pura Raza Española breed using REML and ssGREML methods.

	REML			ssGREML		
Trait	$\sigma_{a}^{2}$	$\sigma_{e}^{2}$	$h^{2}$ (SE)	$\sigma_{a}^{2}$	$\sigma_{e}^{2}$	$h^{2}$ (SE)
AFF	0.124	0.360	0.256 (0.010)	0.128	0.357	0.264 (0.010)
ALF	1.270	2.366	0.349 (0.010)	1.291	2.346	0.355 (0.010)
AIF	10.360	97.140	0.096 (0.008)	10.730	96.854	0.100 (0.008)
FN	0.835	5.289	0.136 (0.007)	0.866	5.268	0.141 (0.007)
IF12	12.030	159.780	0.070 (0.007)	12.420	159.400	0.072 (0.007)
PL	0.111	0.443	0.200 (0.012)	0.114	0.442	0.205 (0.012)
RE	49.310	167.800	0.227 (0.010)	50.761	166.470	0.234 (0.010)

$\sigma_{a}^{2}$: additive genetic variance; $\sigma_{e}^{2}$: residual variance; $h^{2}$: heritability; SE: standard error; AFF: age at first foaling; ALF: age at last foaling; AIF: average interval between foalings; FN: total number of foalings; IF12: interval between first and second foaling; PL: productive life; RE: reproductive efficiency.

**Table 3:** Comparison of reliabilities between the REML and ssGREML methods for fertility traits in the Pura Raza Española horse breed.

Trait	REML	ssGREML	Increase (%)
AFF	0.461	0.475	3.00
ALF	0.487	0.498	2.32
AIF	0.281	0.290	2.98
FN	0.356	0.369	3.71
IF12	0.244	0.249	2.20
PL	0.301	0.311	3.56
RE	0.406	0.419	3.13

R^2^: reliability; AFF: age at first foaling; ALF: age at last foaling; AIF: average interval between foalings; FN: total number of foalings; IF12: interval between first and second foaling; PL: productive life; RE: reproductive efficiency.

**Table 4:** Comparison of reliabilities between the REML and ssGREML methods for fertility traits based on different criteria in the Pura Raza Española horse breed.

	Criteria							
	Sex		Number of Stallions’ Foals		Genotyped		Initial REML Reliability	
Trait	Stallions	Mares	≥40	<40	No	Yes	≥0.6	<0.6
AFF	4.63	2.73	0.75	3.21	1.83	18.90	−0.03	2.45
ALF	4.24	2.00	0.51	2.81	1.19	17.81	−0.36	2.30
AIF	4.57	2.68	1.76	3.42	1.55	22.20	0.00	3.08
FN	5.24	3.44	1.38	3.94	2.34	21.98	−0.03	3.26
IF12	3.89	1.87	2.00	2.85	0.80	21.00	0.00	2.63
PL	4.96	3.30	3.16	3.81	2.25	24.04	0.00	3.84
RE	4.84	2.83	0.96	3.47	1.83	20.72	0.13	3.08

AFF: age at first foaling; ALF: age at last foaling; AIF: average interval between foalings; FN: total number of foalings; IF12: interval between first and second foaling; PL: productive life; RE: reproductive efficiency.

## Data Availability

The data supporting the findings of this study are the property of the Royal National Association of Spanish Horse Breeders (ANCCE). Access to the data for scientific purposes may be requested directly from the breeder’s association (mejoragenetica@lgancce.es).
